# Differences in the Prevalence and Impact of Arthritis Among Racial/Ethnic Groups in the United States, National Health Interview Survey, 2002, 2003, and 2006

**Published:** 2010-04-15

**Authors:** Julie Bolen, Linda Schieb, Jennifer M. Hootman, Charles G. Helmick, Kristina Theis, Louise B. Murphy, Gary Langmaid

**Affiliations:** Centers for Disease Control and Prevention, Division of Adult and Community Health; Centers for Disease Control and Prevention, Atlanta, Georgia; Centers for Disease Control and Prevention, Atlanta, Georgia; Centers for Disease Control and Prevention, Atlanta, Georgia; Centers for Disease Control and Prevention, Atlanta, Georgia; Centers for Disease Control and Prevention, Atlanta, Georgia; Northrop Grumman, Atlanta, Georgia

## Abstract

We describe the prevalence of doctor-diagnosed arthritis and its impact on activities, work, and joint pain for 6 racial/ethnic groups: non-Hispanic whites, non-Hispanic blacks, Hispanics, American Indians/Alaska Natives, Asians and Pacific Islanders, and multiracial or "other" respondents. We combined data from the 2002, 2003, and 2006 National Health Interview Survey (n = 85,784) and, after adjusting for age, sex, and body mass index, compared racial/ethnic differences. Arthritis-attributable activity limitation, arthritis-attributable work limitation, and severe joint pain were higher for non-Hispanic blacks, Hispanics, and multiracial or other respondents with arthritis compared with non-Hispanic whites with arthritis. Our finding that arthritis disproportionately affects certain racial/ethnic minorities may be useful for planning interventions.

## Objective

Arthritis affects 1 in 5 adults and is the most common cause of disability in the United States (1,2). It interferes with work and daily activities (3) and complicates the management of other chronic diseases (4,5). The prevalence and impact of arthritis (3,6) have been explored for large racial/ethnic groups (non-Hispanic blacks and whites, Hispanics, and non-Hispanic "other" races) but not among smaller non-Hispanic groups, which is necessary to tailor arthritis interventions appropriately. We update and expand estimates of the prevalence of arthritis and its impact on activities, work, and joint pain for 6 racial/ethnic groups.

## Methods

We combined data from the 2002, 2003, and 2006 National Health Interview Survey (NHIS), an in-person nationally representative survey of the US civilian, noninstitutionalized population aged 18 years or older. We did not include NHIS data for 2004 and 2005 because arthritis-attributable work limitation and joint pain were not assessed in those years. Doctor-diagnosed arthritis was defined as answering yes to the question, "Have you ever been told by a doctor or other health professional that you have some form of arthritis, rheumatoid arthritis, gout, lupus, or fibromyalgia?" Among respondents with arthritis, arthritis-attributable activity limitation was defined as answering yes to the question, "Are you now limited in any way in any of your usual activities because of arthritis or joint symptoms?" For adults aged 18 to 64 years, arthritis-attributable work limitation was defined as answering yes to the question, "Do arthritis or joint symptoms now affect whether you work, the type of work you do, or the amount of work you do?" Respondents were asked to assess their joint pain in the past 30 days, on average, on a scale of 0 to 10, "where 0 is no pain or aching and 10 is pain or aching as bad as it can be." Severe joint pain was defined as 7 to 10. We present weighted estimates for non-Hispanic whites ("whites"), non-Hispanic blacks ("blacks"), Hispanics, American Indians/Alaska Natives (AI/AN), Asians and Pacific Islanders (API), and multiracial or "other" respondents (MRO). People who reported their ethnicity as Hispanic were not further classified by race in this study. We calculated crude and age-adjusted prevalence estimates, odds ratios with logistic regression, and 95% confidence intervals by using SUDAAN, version 10.0 (RTI International, Research Triangle Park, North Carolina) to account for the multistage probability sample. We age-adjusted estimates to the 2000 US population (7). We used *t* tests for differences in proportions and Wald *F* tests for differences in odds ratios. Differences were considered significant at *P* < .05.

## Results

The sample size was 85,784, a mean response rate of 74%. The annualized prevalence of doctor-diagnosed arthritis was 21% (Table 1). The estimated unadjusted prevalence and weighted population for each group was whites, 24% (36 million); blacks, 19% (4.6 million); Hispanics, 11% (2.9 million); AI/AN, 25% (280,000); API, 8% (667,000); and MRO, 21% (469,000). Arthritis prevalence was significantly higher among respondents aged 45 years or older, women, obese respondents, and respondents with less education (Table 1).

Of adults who reported arthritis, 38% reported activity limitation, 31% (of respondents aged 18-64 years) reported work limitation, and 26% reported severe joint pain during the preceding 30 days (Table 2). Differences in these prevalences were observed among the 6 racial/ethnic groups. The prevalence of arthritis was significantly lower among Hispanics and API than among whites and significantly higher among AI/AN. The prevalences of activity limitation, work limitation, and severe joint pain were significantly higher among blacks, Hispanics, and MRO than among whites.

Results of logistic regression analyses indicate that after adjusting for age, sex, and body mass index, API, Hispanics, and blacks were less likely and AI/AN were more likely than whites to have arthritis (Figure). Blacks and Hispanics were approximately 1.3 times as likely as whites to have activity limitation, 1.8 to 1.9 times as likely to have severe joint pain, and 1.6 to 1.7 times as likely to have work limitation. MRO were 1.7 times as likely as whites to report activity limitation, 1.9 times as likely to report severe joint pain, and 2.2 times as likely to report work limitation. Differences between whites and AI/AN and between whites and API for all 3 impact measures were not significant, although statistical power was limited by small sample sizes.

**Figure.**
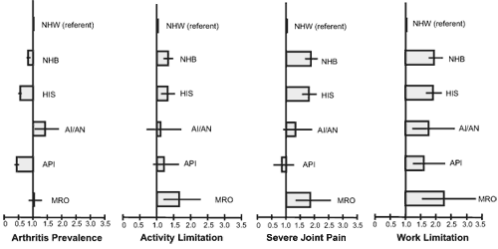
Odds of having arthritis and associated limitations, by race/ethnicity, National Health Interview Survey, United States, 2002, 2003, and 2006. Odds ratios were adjusted for age, sex, and body mass index. The horizontal lines indicate confidence intervals. Abbreviations: NHW, non-Hispanic white; NHB, non-Hispanic black; HIS, Hispanic; AI/AN, American Indian/Alaska Native; API, Asian/Pacific Islander; MRO, multiracial or "other." Data for other and multiple race populations are combined. Respondents in this category were non-Hispanic. Arthritis was defined as answer of yes to the question, "Have you ever been told by a doctor or other health professional that you have some form of arthritis, rheumatoid arthritis, gout, lupus, or fibromyalgia?" Arthritis-attributable activity limitation was defined as answer of yes to the question, "Are you now limited in any way in any of your usual activities because of arthritis or joint symptoms?" Severe joint pain was defined as a rating of 7-10 on a scale assessing average pain in the last 30 days, with 0 being no pain and 10 being worst pain. (Approximately 28% of respondents with doctor-diagnosed arthritis did not report joint pain in the past 30 days and were not asked the question about pain severity. For this analysis, these respondents were classified as not having severe joint pain and were included in the denominator.) Arthritis-attributable work limitation was defined as answer of yes to the question, "Do arthritis or joint symptoms now affect whether you work, the type of work you do, or the amount of work you do?" among working-age respondents (18-64 years).

**Table:** 

**Category**	**Race/Ethnicity, Odds Ratio (95% CI)^a^**					

	**Non-Hispanic White**	**Non-Hispanic Black**	**Hispanic**	**American Indian/Alaska Native**	**Asian/Pacific Islander**	**Multiracial or “Other”^b^**
Arthritis prevalence^c^	1 [Reference]	0.83 (0.78-0.89)	0.54 (0.50-0.58)	1.42 (1.07-1.88)	0.42 (0.35-0.50)	1.00 (0.85-1.29)
Activity limitation^d^	1 [Reference]	1.33 (1.20-1.46)	1.31 (1.14-1.51)	1.10 (0.70-1.71)	1.21 (0.89-1.64)	1.66 (1.21-2.27)
Severe joint pain^e^	1 [Reference]	1.87 (1.67-2.08	1.80 (1.57-2.05)	1.32 (0.91-1.90)	0.84 (0.57-1.25)	1.85 (1.34-2.55)
Work limitation^f^	1 [Reference]	1.65 (1.45-1.89)	1.59 (1.35-1.86)	1.33 (0.81-2.18)	1.04 (0.64-1.69)	2.22 (1.51-3.26)

Abbreviation: CI, confidence interval.

a Odds ratios were adjusted for age, sex, and body mass index.

b Data for other and multiple race populations are combined. Respondents in this category were non-Hispanic.

c Answer of yes to the question, “Have you ever been told by a doctor or other health professional that you have some form of arthritis, rheumatoid arthritis, gout, lupus, or fibromyalgia?”

d Answer of yes to the question, “Are you now limited in any way in any of your usual activities because of arthritis or joint symptoms?”

e Rating of 7-10 on a scale assessing average pain in the last 30 days, with 0 being no pain and 10 being worst pain. (Approximately 28% of respondents with doctor-diagnosed arthritis did not report joint pain in the past 30 days and were not asked the question about pain severity. For this analysis, these respondents were classified as not having severe joint pain and were included in the denominator.)

f Among working-age respondents (18-64 years), answer of yes to the question, “Do arthritis or joint symptoms now affect whether you work, the type of work you do, or the amount of work you do?”

## Discussion

Arthritis affects some racial/ethnic populations disproportionately. For example, the prevalence of arthritis is lower among blacks and Hispanics than among whites, but impact is worse. We generated estimates for smaller racial/ethnic groups that had been combined in a "non-Hispanic other" category previously (3). Separating these groups reveals differences such as a higher prevalence of arthritis among AI/AN and MRO compared with whites and API. Our findings can be used to develop effective and culturally sensitive interventions that can be tailored to these populations.

Reasons for racial/ethnic differences are unknown and merit further investigation. They may be related to health care access, use of health care services, language barriers (8), differences in the prevalence of risk factors for arthritis and related disability (eg, obesity, physically demanding jobs), and cultural differences in the understanding of survey questions, willingness to report limitation and pain, and variations in patterns of medication use and self-management approaches to manage pain.

Our findings are subject to the following limitations: 1) arthritis was self-reported and unconfirmed by a doctor, although the case-finding question appears to be valid for surveillance purposes (9); 2) Hispanics and blacks report less access to health care than do whites and may be less likely to be diagnosed with arthritis, resulting in an underestimate of the prevalence of arthritis in these populations (10); and 3) the small number of AI/AN, API, and MRO in the sample limited our ability to precisely estimate the impact of arthritis. However, all estimates meet data quality standards set for the NHIS (<30% relative standard error). The results for the MRO group should be interpreted with caution, because it is a heterogeneous group. Strengths of the study include its large sample size, impact measures important to people with arthritis (eg, pain severity), and data to classify respondents into multiple race and ethnicity categories.

The US population is becoming more diverse. As data accumulate on the racial/ethnic differences in arthritis prevalence and impact, we need to develop culturally appropriate interventions for the populations most affected. Evidence-based public health interventions that improve pain and functional limitations reach few adults with arthritis (11). Future efforts to increase reach should use appropriately tailored interventions such as the Spanish-language health communication campaign *Buenos Dias Artritis* (12).

## Figures and Tables

**Table 1:** Prevalence of Arthritis Among Adults,^a^ by Race/Ethnicity, National Health Interview Survey, United States, 2002, 2003, and 2006

**Characteristic**	Total, % (95% CI) N = 85,784	White^b^, % (95% CI) n = 54,493	Black^b^, % (95% CI) n = 12,063	Hispanic, % (95% CI) n = 14,880	AI/AN, % (95% CI) n = 391	API, % (95% CI) n = 3,009	MRO^c^, % (95% CI) n = 948
**Overall prevalence**							
Unadjusted	21.2 (20.8-21.6)	23.8 (23.4-24.3)	19.4 (18.5-20.4)	11.1 (10.5-11.8)	25.2 (20.8-30.2)	8.4 (7.2-9.8)	20.7 (17.7-24.0)
Adjusted^d^	21.1 (20.7-21.4)	22.3 (21.9-22.7)	21.8 (21.0-22.7)	15.6 (14.7-16.4)	28.6 (24.5-33.1)	10.6 (9.2-12.2)	23.6 (20.7-26.7)
**Prevalence by subgroup**							
**Age, y**							
18-44	7.5 (7.2-7.9)	8.9 (8.5-9.4)	6.7 (6.0-7.5)	3.6 (3.1-4.0)	14.5 (9.1-22.4)	2.2 (1.6-2.9)	10.4 (7.9-13.5)
45-64	29.2 (28.6-29.9)	30.6 (29.9-31.4)	30.3 (28.6-32.0)	22.0 (20.3-23.9)	38.1 (30.1-46.7)	12.0 (9.6-15.0)	33.5 (27.2-40.4)
≥65	48.8 (47.8-49.7)	49.2 (48.1-50.3)	54.0 (51.5-56.4)	41.6 (38.4-44.8)	55.6 (38.4-71.5)	34.6 (28.4-41.3)	47.5 (38.4-56.7)
**Sex**							
Men	17.4 (16.9-17.9)	20.2 (19.6-20.8)	14.0 (12.8-15.3)	7.7 (7.0-8.5)	22.3 (15.7-30.6)	6.7 (5.3-8.3)	14.5 (11.3-18.4)
Women	24.7 (24.2-25.2)	27.2 (26.6-27.9)	23.8 (22.7-25.0)	14.6 (13.7-15.6)	28.0 (20.6-36.9)	10.1 (8.2-12.3)	26.2 (22.1-30.8)
**Body mass index**							
<25.0 kg/m^2^	15.9 (15.4-16.4)	18.1 (17.5-18.8)	13.5 (12.3-14.7)	7.0 (6.2-7.8)	16.3 (10.1-25.1)	7.0 (5.8-8.6)	15.4 (11.4-20.5)
25.0-29.9 kg/m^2^	21.1 (20.5-21.7)	24.0 (23.3-24.7)	16.8 (15.4-18.4)	11.4 (10.4-12.5)	28.8 (20.4-39.1)	9.9 (7.6-12.7)	15.5 (11.6-20.3)
≥30.0 kg/m^2^	30.5 (29.7-31.3)	33.9 (32.8-34.9)	27.2 (25.5–29.0)	17.2 (15.8-18.7)	30.4 (23.4-38.5)	17.7 (11.9-25.4)	33.2 (26.8-40.3)
**Education**							
Less than high school	25.2 (24.3-26.2)	31.7 (30.3-33.1)	31.6 (29.5-33.9)	12.2 (11.3-13.2)	27.2 (17.2-40.1)	17.4 (12.9-23.0)	25.4 (18.2-34.1)
High school diploma	23.2 (22.6-23.9)	26.3 (25.5-27.1)	18.2 (16.8-19.6)	10.6 (9.4-11.8)	21.2 (13.9-31.0)	8.5 (5.8-12.2)	18.3 (13.2-24.7)
More than high school	18.9 (18.5-19.4)	21.0 (20.4-21.6)	14.9 (13.7-16.1)	10.2 (9.1.-11.4)	26.9 (20.0-35.2)	6.6 (5.4-8.0)	20.8 (17.0-25.1)

Abbreviations: CI, confidence interval; AI/AN, American Indian/Alaska Native; API, Asian/Pacific Islander; MRO, multiracial or other.

a Answer of yes to the question, "Have you ever been told by a doctor or other health professional that you have some form of arthritis, rheumatoid arthritis, gout, lupus, or fibromyalgia?"

b Non-Hispanic.

c Data for "other" and multiple race populations are combined. Respondents in this category were non-Hispanic.

d Adjusted for age with the 2000 census projected population (7).

**Table 2:** Proportion of Adults With Arthritis^a^ Who Have Arthritis-Attributable Activity Limitation, Severe Joint Pain, or Arthritis-Attributable Work Limitation, by Race/Ethnicity, National Health Interview Survey, United States, 2002, 2003, and 2006

Characteristic	Total, % (95% CI) N = 85,784	White^b^, % (95% CI) n = 54,493	Black^b^, % (95% CI) n = 12,063	Hispanic, % (95% CI) n = 14,880	AI/AN, % (95% CI) n = 391	API, % (95% CI) n = 3,009	MRO^c^, % (95% CI) n = 948
**Activity limitation^d^**							
Unadjusted	37.7 (36.9-38.6)	36.2 (35.2-37.3)	44.6 (42.6-46.7)	43.2 (40.2-46.3)	39.1 (29.0-50.2)	38.2 (31.8-45.1)	49.5 (41.8-57.2)
Adjusted^e^	35.8 (34.8-36.9)	34.3 (33.0–35.6)	43.3 (40.4-46.2)	41.7 (37.9-45.5)	31.6 (24.1-40.2)	32.2 (24.7-40.8)	47.6 (39.1-56.1)
**Severe joint pain^f^**							
Unadjusted	25.6 (24.9-26.4)	23.1 (22.3-24.0)	38.3 (36.0-40.5)	36.4 (33.8-39.1)	28.7 (21.9-36.6)	18.5 (13.4-25.0)	36.6 (29.7-44.1)
Adjusted	25.4 (24.3-26.5)	23.0 (21.8-24.3)	36.7 (33.8-39.8)	35.5 (32.1-39.0)	26.5 (20.0-34.2)	17.8 (12.3-25.0)	33.7 (26.5-41.8)
**Work limitation^g^**							
Unadjusted	31.2 (30.2-32.3)	28.8 (27.6-30.0)	41.6 (38.9-44.4)	39.7 (36.4-43.1)	37.7 (27.3-49.4)	28.2 (19.9-38.2)	46.4 (37.6-55.4)
Adjusted	30.8 (29.6-32.1)	28.6 (27.1-30.2)	40.8 (37.3-44.3)	38.7 (34.9-42.7)	30.2 (21.9-40.0)	27.0 (18.4-37.9)	42.7 (33.4-52.6)

Abbreviations: CI, confidence interval; AI/AN, American Indian/Alaska Native; API, Asian/Pacific Islander; MRO, multiracial or other.

a Answer of yes to the question, "Have you ever been told by a doctor or other health professional that you have some form of arthritis, rheumatoid arthritis, gout, lupus, or fibromyalgia?"

b Non-Hispanic.

c Data for "other" and multiple race populations are combined. Respondents in this category were non-Hispanic.

d Answer of yes to the question, "Are you now limited in any way in any of your usual activities because of arthritis or joint symptoms?"

e Adjusted for age with the 2000 census projected population (7).

f Rating of 7-10 on a scale assessing average pain in the last 30 days, with 0 being no pain and 10 being worst pain. (Approximately 28% of respondents with doctor-diagnosed arthritis did not report joint pain in the past 30 days and were not asked the question about pain severity. For this analysis, these respondents were classified as not having severe joint pain and were included in the denominator.)

g Among working-age respondents (18-64 years), answer of yes to the question, "Do arthritis or joint symptoms now affect whether you work, the type of work you do, or the amount of work you do?"
